# Intersectional analysis of social disparities in type 2 diabetes risk among adults in Germany: results from a nationwide population-based survey

**DOI:** 10.1186/s12889-024-17903-5

**Published:** 2024-02-16

**Authors:** Francesca Färber, Enrique Alonso-Perez, Christin Heidemann, Yong Du, Gertraud Stadler, Paul Gellert, Julie Lorraine O’Sullivan

**Affiliations:** 1 Institute of Medical Sociology and Rehabilitation Science, Charité– Universitätsmedizin Berlin, Corporate Member of Freie Universität Berlin and Humboldt- Universität zu Berlin, Charitéplatz 1, 10117 Berlin, Germany; 2 Einstein Center Population Diversity (ECPD) , Berlin, Germany; 3 Department of Epidemiology and Health Monitoring, Robert Koch Institute, General-Pape-Str. 62-66, 12101 Berlin, Germany; 4 Institute of Gender in Medicine (GiM), Charité– Universitätsmedizin Berlin, corporate member of Freie Universität Berlin and Humboldt- Universität zu Berlin, Augustenburger Platz 1 , 13353 Berlin, Germany; 5 German Center for Mental Health (DZPG), Partner Site Berlin/Potsdam, Berlin, Germany

**Keywords:** Diabetes mellitus, Diabetes risk, Prevention, Intersectionality, Social determinants, Population-based survey

## Abstract

**Background:**

Differences in type 2 diabetes risk have been reported for several sociodemographic determinants including sex/gender or socioeconomic status. From an intersectional perspective, it is important to not only consider the role of social dimensions individually, but also their intersections. This allows for a deeper understanding of diabetes risk and preventive needs among diverse population groups.

**Methods:**

As an intersectionality-informed approach, multilevel analysis of individual heterogeneity and discriminatory accuracy (MAIHDA) was used in a population-based sample of adults without known diabetes in Germany from the cross-sectional survey “Disease knowledge and information needs– Diabetes mellitus (2017)”. Diabetes risk was assessed by the German Diabetes Risk Score (GDRS, range 0-122 points), estimating the individual risk of developing type 2 diabetes within the next 5 years based on established self-reported risk factors. Nesting individuals in 12 intersectional strata defined by combining sex/gender, educational level, and history of migration, we calculated measures to quantify the extent to which individual differences in diabetes risk were explained at strata level, and how much this was due to additive or multiplicative intersectional effects of social determinants.

**Results:**

Drawing on data of 2,253 participants, we found good discriminatory accuracy of intersectional strata (variance partition coefficient = 14.00% in the simple intersectional model). Model-predicted GDRS means varied between 29.97 (corresponding to a “low risk” of < 2%) in women with high educational level and a history of migration, and 52.73 (“still low risk” of 2–5%) in men with low educational level without a history of migration. Variance in GDRS between strata was mainly explained by additive effects of social determinants (proportional change in variance to intersectional interaction model = 77.95%) with being male and having low educational level being associated with higher GDRS. There was no evidence of multiplicative effects in individual strata.

**Conclusions:**

Type 2 diabetes risk differed between intersectional strata and can to some extent be explained at strata level. The role of intersectional effects was minor and needs to be further investigated. Findings suggest a need for specific preventive measures targeted at large groups with increased diabetes risk, such as men and persons with low educational level.

**Supplementary Information:**

The online version contains supplementary material available at 10.1186/s12889-024-17903-5.

## Background

Diabetes mellitus is a noncommunicable disease with high relevance for public health in many countries around the world. Globally, more than half a billion people were estimated to have diabetes mellitus, with type 2 diabetes accounting for more than 90% of all cases [1]. For Germany, the 12-months prevalence of self-reported diabetes in adults was estimated at 8.9% in 2019/2020 [2]. Diabetes represents one of the most important causes of burden of disease in terms of disability-adjusted life years in Germany, largely due to type 2 diabetes [3] causing approximately 7.4 billion euros in direct medical costs per year [4].

The global increase in age-standardized incidence and prevalence over the last decades is considered to be mainly due to changes in behavioural and environmental risk factors [5]. These include aspects like poor dietary patterns, low levels of physical activity and increased sedentary time, all associated with obesity as a main risk factor of type 2 diabetes besides age [6, 7]. These behavioural aspects are potentially modifiable and can therefore be targeted by preventive measures.

Importantly from a public health perspective, there is consistent evidence for social inequalities in the occurrence of diabetes and its risk factors, which can guide prevention approaches. There are sex/gender differences in diabetes prevalence, with more men being affected than women, both globally (9.0% vs. 7.9% [8]), as well as in Germany (9.6% vs. 8.2%, [2]). These sex/gender differences also extend to risk factors [9]. While men were less likely to lead a health-promoting lifestyle considering aspects like smoking or fruit and vegetable intake, they showed higher levels of physical activity compared to women [10].

Moreover, low socioeconomic status, typically indicated by low educational level, low income, or less qualified occupation, has consistently been associated with a higher risk of developing diabetes [11, 12]. In Germany [13], educational level was found to be a more pronounced predictor for diabetes compared to income and occupation [14, 15]. In addition, there is considerable evidence of socioeconomic inequality across diverse risk factors for diabetes including obesity, smoking, or physical inactivity, which are more prevalent among people with low socioeconomic status [10, 16].

Further, studies with European populations have reported a higher diabetes prevalence and diabetes-related mortality for persons with a history of migration compared to those without a history of migration, with the degree of risk varying depending on the region from which people have migrated [17, 18]. In Germany, health data on people with a history of migration has long been insufficient [19]. Analyses in this field face challenges, as public health data often contain little information on history of migration [20, 21] and differ in their definition of migration-related variables [21]. Consequently, there is a need to investigate the relevance of migration history for the risk of developing diabetes among the German population.

Given the findings on the role of individual social determinants, public health researchers have argued that health inequalities need to be examined beyond single axes of social dimensions. This lead to proposing an intersectional perspective that takes into account the complex interplay of such determinants, and the differential effects of social positions at the unique intersections of those dimensions [22]. Intersectionality, as conceptualized by Black feminist scholars (e.g., Crenshaw [23]), is thus considered a valuable framework for public health research [24]. Intersectional theory assumes that dimensions of social position such as sex/gender or socioeconomic status intersect at the individual level and jointly shape a person’s experience in ways that reflect systems of privilege and oppression at the structural level [24, 25]. Moreover, the advantages and disadvantages that result from a particular social position, for example in relation to health, do not correspond to a simple additive accumulation of the effects of intersecting dimensions, but can be characterized by differential multiplicative intersectional effects [22, 26].

In epidemiological research on diabetes, Wemrell et al. [27] applied an intersectional perspective to analyse disparities in diabetes risk using Swedish registry data of the population aged 40 and older. They found a heterogeneous distribution of diabetes prevalence across social dimensions such as age, gender, income, education, and migration status. For instance, elderly migrated men with low income and low education levels had a relatively high risk of type 2 diabetes, while women who did not migrate, aged 40–49 years old, with high income and high education levels had a relatively low risk. Multilevel analysis of individual heterogeneity and discriminatory accuracy (MAIHDA) has been increasingly proposed as an appropriate intersectionality-informed approach for the quantitative study of health inequalities [22, 28]. Essentially, in MAIHDA individuals are located within strata based on their combination of social dimension characteristics and in a next step, health outcomes of interest are modelled using multilevel regression, with individuals considered to be nested in those intersectional strata [29]. According to Evans et al. [30], the aims of the MAIHDA approach are threefold: (1) to map differences in health outcomes across intersectional strata by estimating means or frequencies for all strata, (2) to quantify the variance within and between strata by calculating measures indicating discriminatory accuracy of intersectional strata, and (3) to estimate multiplicative intersectional effects for all strata.

Holman et al. [31] applied MAIHDA to map intersectional inequalities in biomarkers using English national data including HbA1c, a measure of blood glucose concentration used to diagnose diabetes. Examining intersectional strata defined by the combination of gender, ethnicity, education, and income, they found some between-strata variance for HbA1c with lowest levels in White women with high education and high income, and highest levels in Black and Minority Ethnicity men with low education and low income.

Building on this first evidence, we aimed to investigate inequalities in diabetes risk in Germany by adopting an intersectional perspective. To this end, we employed the MAIHDA approach using data on diabetes risk based on health behaviour and other risk factors from a sample of persons without known diabetes from a nationwide population-based survey in Germany to answer the following questions:

1. To what extent does diabetes risk vary between individuals from different intersectional strata defined by sex/gender, educational level, and history of migration?
2. To what extent do differences in diabetes risk across intersectional strata result from additive main effects and from multiplicative intersectional effects of the social dimensions defining these strata?

## Methods

### Study design and sample

In this study, we used data from the nationwide telephone interview survey “Disease knowledge and information needs– Diabetes mellitus (2017)”, conducted by the Robert Koch Institute (RKI) in cooperation with the Office for National Education and Communication on Diabetes Mellitus of the German Federal Centre for Health Education (BZgA), and the Institute of Medical Sociology and Rehabilitation Science of the Charité– Universitätsmedizin Berlin. The survey and its sampling procedure [32, 33], as well as psychometric properties of multi-item scales applied in the study [34] have been previously described in more detail elsewhere.

The target population were adults (≥ 18 years) with sufficient German language skills to participate in the standardized interview. Computer-assisted telephone interviews were carried out by trained personnel from a market and social research institute from September to December 2017 using a dual frame approach. A sample of landline and cell phone numbers was randomly generated with the aim of providing a representative sample of all private households reachable by phone at a national level. Sampling proceeded in two steps, beginning with the drawing of a sample from the general adult population and applying the Kish selection grid to randomly select members from multi-person households. Depending on their response to the question “Have you ever been diagnosed with diabetes by a doctor?” (“yes” or “no”), participants were interviewed based on a questionnaire for individuals with or without a diagnosis of diabetes. This phase resulted in the inclusion of 263 persons with and 2,327 persons without self-reported diabetes. The response rate was 17.9%, calculated according to the standard of the American Association for Public Opinion Research as proportion of realized interviews in relation to all households potentially reachable by phone in Germany, i.e., response rate 3 [33]. In a second sampling phase with direct screening for persons with diagnosed diabetes, 1,216 adults with diabetes were included, resulting in a study sample of 3,806 people in total (2,327 individuals without diabetes and 1,479 individuals with diabetes).

Because of the preventive perspective of the present study, only individuals without diabetes were considered. From the total number of 2,327 participants without known diabetes, 7 persons (0.30%) with missing data for educational level or history of migration were excluded from the analysis. Furthermore, values for diabetes risk could not be calculated for 67 persons (2.88%), because of missing data regarding components of the diabetes risk score. Thus, models were estimated based on data of 2,253 persons.

Prior to the interview, all participants were informed about the procedure of the interview, the aims of the survey, including data analyses in anonymized form in studies to improve health information and preventive services for the population, the voluntary nature of their participation, as well as data protection regulations, and gave verbal informed consent to participate. The “Disease knowledge and information needs– Diabetes mellitus (2017)” survey was approved by the ethics committee of the Berlin Chamber of Physicians in August 2017 (Ärztekammer Berlin; No. Eth-23/17) and the Federal Commissioner for Data Protection and Freedom of Information.

### Variables

#### Outcome

The outcome of interest was diabetes risk assessed by the German Diabetes Risk Score (GDRS) developed by the German Institute of Human Nutrition Potsdam-Rehbrücke (DIfE) [35, 36]. The score was derived based on data from the European Prospective Investigation into Cancer and Nutrition (EPIC)-Potsdam study [35] and enables to predict the individual risk for developing type 2 diabetes within the next 5 years based on non-invasively assessed factors. The GDRS is an established and validated tool that has been shown to accurately predict 5-year-risk of type 2 diabetes [35, 37, 38]. In this study, an updated and simplified version of the GDRS [39] was used as validated in the population-based German Health Interview and Examination Survey for Adults (2008–2011) [38]. The score is calculated by assigning points to the following risk factors as categorical score components: age, waist circumference, body height, prevalent hypertension, smoking, physical activity, coffee consumption, whole grain intake, meat intake and family history of diabetes. Details on the calculation can be found elsewhere [39]. GDRS values can range from 0 to 122. According to the DIfE recommendations on the communication of individual risks based on GDRS results, a score < 46 points can be interpreted as low risk (corresponding to a risk < 2%), 46–56 points as still low risk (≈ 2–5%), 57–63 points as elevated risk (≈ 6–10%), and a score > 63 points as high to very high risk (> 10%) of developing type 2 diabetes within the next 5 years [36].

In the “Disease knowledge and information needs– Diabetes mellitus (2017)” survey, prevalent hypertension was assessed by self-report on physician-diagnosed hypertension. Smoking was measured by asking whether participants currently or formerly smoked, and the average number of cigarettes, cigarillos or cigars smoked daily (< 20 or ≥ 20). Physical activity was assessed by determining whether a person was physically active for at least 5 h per week or not. Coffee consumption was measured in cups of coffee per day. Whole grain intake was assessed by the average sum of whole grain slices and muesli portions consumed daily. Red meat intake was obtained by asking how frequently a person consumed beef, pork, or lamb. Family history of diabetes was assessed by self-report on diagnoses of diabetes in biological parents and siblings. Since waist circumference was not assessed in the interview, it was estimated based on information regarding height, weight and age using separate equations for men and women (see Heidemann et al. [40] for more detail). Equations were obtained from data of the German Health Interview and Examination Survey for Adults (2008–2011) that provided comprehensive information on measured and self-reported anthropometric variables [41].

#### Intersectional variables

The social dimensions examined in this study were sex/gender, educational level, and history of migration. In the survey, sex/gender was assessed by categories “male” and “female” as response options to the question “Are you male/female?“. Educational level was measured by the Comparative Analysis of Social Mobility in Industrial Nations classification (CASMIN) using a categorization into “low”, “middle” and “high educational level” as follows: incomplete general education, general elementary education or basic vocational qualification were classified as “low”; intermediate general education or vocational qualification or full maturity certificates were classified as “middle”; and lower or higher tertiary education were classified as “high educational level” [42]. History of migration was defined as having at least one parent who was not born in Germany. Consequently, this includes persons with a two-sided migration history, i.e., participants whose parents were both not born in Germany, or those who were not born in Germany themselves and of whom at least one parent was not born in Germany. Otherwise, participants were classified as “not having a history of migration”.

By combining all possible categories of sex/gender, educational level, and history of migration, 12 intersectional strata were obtained (2 × 3 × 2). The classifications described above were selected with the aim to investigate relevant strata building on the current state of research on social determinants of diabetes risk, while accounting for sufficiently large case numbers.

Since the risk of developing diabetes increases with age [1], age is a component of the GDRS used to assess diabetes risk. Therefore, we opted not to include age as a social dimension to construct the intersectional strata. To examine whether age was driving observed effects in relation to included social dimensions, we ran a sensitivity analysis including age in years as a continuous control variable.

### Statistical analyses

As previously described [32], a survey-specific weighting factor was used in all analyses to increase representativeness of results. It was computed to compensate for deviations in the distribution of age, sex/gender, educational level, and federal state of residence in the study sample compared to the resident population in Germany as of December 31, 2016, reported by the Federal Statistical Office. Descriptive statistics were calculated using the R packages “survey” (version 4.1.1; [43]) and “srvyr” (version 1.2.0; [44]), creating a survey design object with the weighting factor.

We performed a MAIHDA [26, 28] running two consecutive multilevel linear regressions to model diabetes risk with individuals at level one nested in 12 intersectional strata at level two. Firstly, as **null model** or **simple intersectional model**, we fitted an **unadjusted random intercepts model** including the intersectional strata as random effects in addition to an intercept.

Model 1 can be denoted as follows:

$$y_{ij}= {\beta}_{0} + u_{j} + e_{ij}$$

with *y*_*ij*_ indicating the diabetes risk score of an individual *i* (*i* = 1,…, *n*_*j*_) in stratum *j* (*j* = 1,…, *J*), β_0_ denoting the intercept, *u*_*j*_ indicating stratum-level random effects assumed to be normally distributed with mean 0 and variance $\sigma$_u_^2^, and *e*_*ij*_ denoting individual-level residuals assumed to be normally distributed with mean 0 and variance $\sigma$_e_^2^. Based on this variance component model, total variance in diabetes risk score is partitioned into variance within intersectional strata $\sigma$_e_^2^ and variance between intersectional strata $\sigma$_u_^2^. The latter is attributable to both additive main effects and possible multiplicative intersectional effects of the individual social dimensions in this unadjusted model.

In a next step, we fitted a **second model, the intersectional interaction model**, **adjusted for the main effects** of the social dimensions constructing the intersectional strata. This was implemented by including variables for sex/gender, educational level, and history of migration as fixed effects in addition to an intercept and the stratum-level random effects as in the null model.

Hence, model 2 can be denoted as follows:

$$y_{ij}= {\beta}_{0} + {\beta}_{1} \times_{1j} + {\beta}_{2} \times _{2j} + {\beta}_{3} \times_{3j} + {\beta}_{4} \times_{4j} + u_{j} + e_{ij}$$

with indicator variables *x*_*1j*_ for male, *x*_*2j*_ for having a history of migration, as well as *x*_*3j*_ for middle and *x*_*4j*_ for low educational level, and corresponding regression coefficients β_1_,…, β_4_. Assumptions on the distribution of stratum-level random effects and individual-level residuals corresponded to those in model 1. The estimation of stratum-level random effects is not affected by which category of a social dimension is chosen as a reference [26, 30]. Adjusting for main effects of social dimensions allows to isolate the proportion of between-strata variance in diabetes risk score that is potentially attributable to stratum specific two-way or higher interactions of those variables, i.e., multiplicative intersectional effects, from the proportion explained by additive main effects.

Consequently, several measures of interest were determined based on these two models:

1. We calculated variance partition coefficients (VPC), which reflect the proportion of total variance in diabetes risk score explained by between-strata variance:

$$ VPC= \frac{{\sigma }_{u}^{2}}{{\sigma }_{u}^{2}+ {\sigma }_{e}^{2} }$$

The VPC is considered a measure of discriminatory accuracy of intersectional strata [28] and corresponds to the intraclass correlation (ICC) in both models. The higher the VPC in model 1, the higher the relevance of intersectional strata in explaining individual differences in diabetes risk scores [45]. The higher the VPC in model 2, the higher the relevance of multiplicative intersectional effects of social dimensions defining the strata in explaining such differences [45].

2. Based on model 1, predicted means for diabetes risk scores and 95% confidence intervals for all intersectional strata were obtained.
3. To quantify the extent to which between-strata variance can be explained by the added fixed effects for the main effects, the proportional change in variance (PCV) was calculated as follows:

$$ PCV= \frac{{\sigma }_{u\left(1\right)}^{2}- {\sigma }_{u\left(2\right)}^{2}}{{\sigma }_{u\left(1\right)}^{2} }$$

It reflects the proportional change in between-strata variance from the simple intersectional model to the intersectional interaction model. The higher the PCV, the higher the proportion of variance in diabetes risk scores between strata that is attributable to additive main effects of social dimensions. Accordingly, 1 - PCV provides information on the relevance of multiplicative intersectional effects in the sense of stratum specific two-way or higher interactions.

4. Further, to examine potential multiplicative intersectional effects within each intersectional stratum, stratum-level residuals and 95% confidence intervals were determined by subtracting the predicted mean based on main effects from the total predicted mean for each stratum based on model 2. Thus, stratum-level residuals reflect the extent to which predicted strata mean values deviate from expected additive main effects of the combination of social dimensions.

Data management was conducted with IBM SPSS Statistics (version 27) and analyses were performed using R statistical software (version 4.2.2, [46]). We estimated all models using a restricted maximum likelihood (REML) procedure with the R package “lme4” (version 1.1.32; [47]) using the “bootpredictlme4” package (version 0.1; [48]) to bootstrap standard errors of predicted means. The weighting factor was rescaled using the “datawizard” package (version 0.7.1; [49]) to estimate the models.

## Results

The 2,253 individuals had a mean age of 51.73 years (SD = 18.59), 50.77% were female and 80.70% had no history of migration (Table 1). According to the CASMIN classification, 26.21% of participants had a high level of education, 40.64% had a middle level and 33.15% had a low level of education.

**Table 1:** Sample characteristics (*n* = 2,253)

Variables	n	Mean (SD) or %
Age (years), mean (*SD*)		51.73 (18.59)
Sex/Gender (%)		
Female	1262	50.77
Male	991	49.23
History of migration (%)		
No history of migration	1912	80.70
History of migration	341	19.30
Educational level (CASMIN) (%)		
High	974	26.21
Middle	956	40.64
Low	323	33.15

Sample sizes (n) are unweighted. Mean (SD) and percentages are weighted

The number of observations per intersectional stratum was higher than 20 in all 12 strata, with the smallest number of observations being 23 for men with low educational level and a history of migration (Table 2).

**Table 2:** Predicted GDRS points with 95% CIs based on model 1 by intersectional stratum

History of migration		Sex/Gender		Educational level			*n*	Predicted GDRS points	(95% CI)
Yes	No	Male	Female	Low	Mid	High			
x		x		x			23	39.08	(26.55, 51.61)
x			x	x			33	40.31	(27.39, 53.23)
x		x			x		59	32.77	(19.54, 45.99)
x			x		x		80	35.80	(22.75, 48.86)
x		x				x	75	37.98	(24.49, 51.46)
x			x			x	71	29.97	(16.81, 43.12)
	x	x		x			110	52.73	(38.90, 66.57)
	x		x	x			157	47.69	(34.79, 60.58)
	x	x			x		323	40.17	(27.08, 53.25)
	x		x		x		494	36.96	(24.11, 49.81)
	x	x				x	401	41.49	(28.41, 54.57)
	x		x			x	427	32.78	(19.78, 45.78)

95% CI = 95% confidence intervals

Table 3 provides results from MAIHDA models 1 and 2. The VPC of the simple intersectional model (unadjusted null model) was 14.00%, indicating the amount of variance in diabetes risk scores at the intersectional strata level. After including fixed effects for sex/gender, history of migration and educational level in the intersectional interaction model, the VPC dropped to 3.09%. The PCV in model 2 was 77.95%, suggesting that differences in diabetes risk scores between intersectional strata resulted mostly from additive effects of the included social dimensions. Accordingly, 22.05% of the between-strata variance in diabetes risk score remained unexplained by additive main effects. Based on fixed effects estimates, diabetes risk was higher for men than for women, lower for people with a history of migration than for people without, as well as higher for persons with low educational level compared to those with high educational level.

**Table 3:** Parameter estimates from MAIHDA intersectional models for diabetes risk score (*n* = 2,253)

	Model 1. Simple intersectional model		Model 2. Intersectional interaction model	
	Estimate	(95% CI)	Estimate	(95% CI)
**Fixed effects**				
Intercept	38.98	(35.04, 42.91)	36.00	(31.69, 40.24)
Sex/Gender				
Female (reference)			-	-
Male			4.18	(0.06, 8.32)
History of migration				
No history of migration (reference)			-	-
History of migration			-5.83	(-10.12, -1.67)
Educational level (CASMIN)				
High (reference)			-	-
Middle			1.03	(-3.79, 5.93)
Low			10.89	(5.74, 16.15)
**Measures of variance**				
Between-strata variance	43.79	(13.79, 94.59)	8.57	(0.25, 28.60)
Within-strata variance	269.00	(247.37, 289.86)	269.09	(247.52, 289.98)
VPC (%)	14.00		3.09	
PCV (%)			77.95	
1-PCV (%)			22.05	

95% CI = 95% confidence intervals; PCV = proportional change in the between-strata variance; VPC = variance partition coefficient

**Fig. 1:**
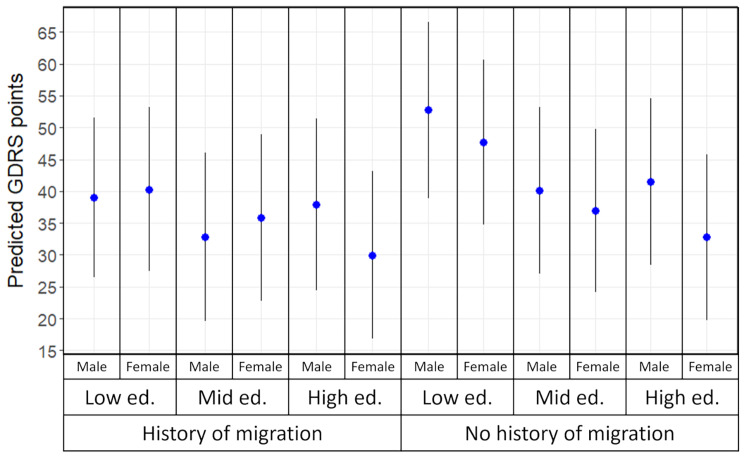
Stratum-specific predicted GDRS points with 95% confidence intervals obtained from model 1. ed.: educational level

As depicted in Fig. 1 and Table 2, predicted means of diabetes risk score based on model 1 varied between intersectional strata. Scores ranged from 29.97 (95% CI: 16.81, 43.12) in women with high educational level and a history of migration, indicating low actual risk, to 52.73 (95% CI: 38.90, 66.57) in men with low educational level and no history of migration, indicating still low actual risk according to the DIfE classification for risk communication based on GDRS [36].

**Fig. 2:**
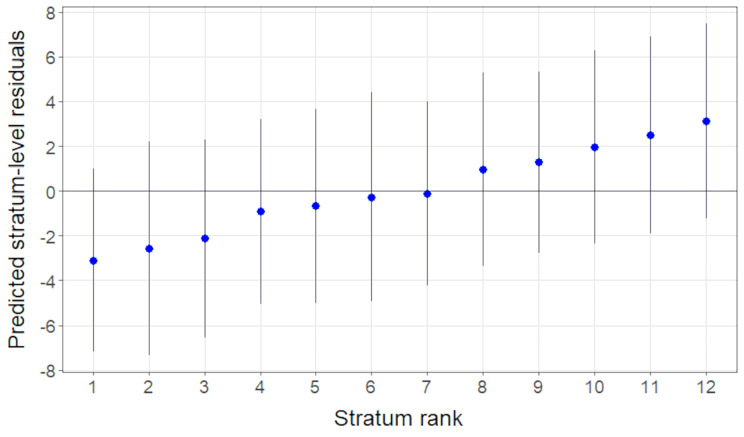
Predicted stratum-level residuals regarding GDRS with 95% confidence intervals obtained from model 2. Stratum ranks were: 1 women with high educational level and no history of migration; 2 men with low educational level and history of migration; 3 men with middle educational level and history of migration; 4 men with middle educational level and no history of migration; 5 women with high educational level and history of migration; 6 women with low educational level and history of migration; 7 women with middle educational level and no history of migration; 8 women with low educational level and no history of migration; 9 men with high educational level and no history of migration; 10 men with low educational level and no history of migration; 11 men with high educational level and history of migration; 12 women with middle educational level and history of migration. (See Additional file 2: Figure S1 for a version with labelling grid sorted by stratum ID)

Stratum-level residuals, i.e., differences between predicted means based on total effects and predicted means based on fixed main effects only, are depicted in Fig. 2. The 95% confidence intervals include 0 for all strata, and thus show no evidence for multiplicative intersectional effects.

As a sensitivity analysis, we fitted models 1 and 2 with an additional control for age. This yielded a slightly lower VPC (model 1: 12.75%; model 2: 0.82%) and a higher PCV (93.55%), thus still following the overall tendencies from the main analysis (see Additional file 1: Table S1). The fixed effect estimates differed from those in the main analysis, in that the main effect of history of migration was not significantly different from 0. Additionally, the effect for mid-level education was significantly different from 0 indicating that individuals with mid-level education had higher diabetes risk scores compared to individuals with high educational level. This result suggests that the main effect of history of migration might be confounded by age.

## Discussion

In the present study, we investigated diabetes risk in persons without known diabetes in Germany with an intersectionality-informed approach. To this end, we used data from a nationwide population-based sample to apply MAIHDA on diabetes risk scores of individuals nested in intersectional strata that were defined by the combination of social dimensions sex/gender, educational level, and history of migration. Multilevel linear regressions allowed us to examine the extent to which individual differences in diabetes risk scores are explained at the strata level, and how much these differences are due to additive main effects or multiplicative intersectional effects of the social determinants.

Model-predicted diabetes risk scores showed disparities among the 12 strata, with differences of approximately 20 points between the stratum with the lowest risk and the one with the highest: The stratum with the lowest risk to develop type 2 diabetes within the next five years consisted of women with high educational level and a history of migration. On the other hand, the stratum comprising men with low educational level and without a history of migration displayed the highest risk. The calculated variance components measure suggested that 14.00% of the individual variance in diabetes risk score could be attributed to between-strata variance. According to Axelsson Fisk et al. [45], this VPC indicates a good level of discriminatory accuracy of intersectional strata, and is rather high compared to other studies applying MAIHDA to health outcomes [50]. Thus, a relevant proportion of the variance in diabetes risk scores can be explained at strata level, highlighting the role of social determinants in the risk of developing type 2 diabetes.

With a PCV of 77.95%, most of the variance explained by intersectional strata could be attributed to the additive main effects of sex/gender, educational level, and history of migration. The directions of the main effects are in line with the existing evidence on sex/gender [9] and educational level [11] as determinants of diabetes risk: being male and having a low educational level was associated with higher risk of developing diabetes compared to being female and having a high educational level, respectively. When controlling for age as part of a sensitivity analysis, mid-level education was also associated with higher diabetes risk scores compared to high educational level, supporting the robust effect of educational level. In contrast, the direction of the main effect of history of migration suggesting a higher risk for individuals without a history of migration, is at odds with studies from other European countries [17] that described a higher risk among individuals with migration history. The result of the present analysis, however, may be due to a confounding of the migration history variable with age: People with a history of migration are on average younger than persons without history of migration in the sample, as well as in German society [51]. As a likely consequence, the fixed effect coefficient for migration history was not significantly different from 0 when controlling for age, unlike the fixed effect coefficients for male sex/gender and low educational level. Further, migration-related disparities in diabetes risk have been attributed to differences in socioeconomic status, lifestyle and health behaviours, biological aspects such as the pathogenesis of insulin resistance, and healthcare access, and thus appear to vary with the region from which people migrated [12, 52, 53]. However, due to the small number of cases, it was not possible to distinguish between regions of origin of the individuals with migration history or between persons with a one- and two-sided history of migration. This could have otherwise clarified the uncertain effect of migration history. Thus, results on history of migration in this analysis should be interpreted with caution.

Given that 22.05% of the between-strata variance in diabetes risk scores remain unexplained by main effects (see 1-PCV), the differences in diabetes risk scores between strata might be attributable, albeit to a relatively lesser extent, to more complex interactions of the defining social dimensions in terms of multiplicative intersectional effects. However, stratum-level residuals for the 12 individual strata revealed no significant deviation of the predicted stratum means from the expected values based on additive main effects of a combination of social dimensions. This could be in part due to the fact that numbers of observations in individual strata were too small to detect multiplicative effects at specific intersections of social dimensions. The partially small group size also did not allow for further differentiation between plausible subgroups, such as regions of migration origin or more differentiated gradations of educational level. Nonetheless, additional subgroups might have explained the differences in model-predicted diabetes risk scores more precisely but had to be collapsed for this study.

Overall, the results are consistent with previous intersectionality-informed findings on diabetes prevalence in Swedish registry data [27] and the previous MAIHDA on HbA1c levels in English national data [31]. These studies found large disparities between intersectional strata, showing that strata consisting of men with low education had less favourable outcomes, while women with high education displayed more favourable outcomes in comparison. However, in line with the body of research already outlined, having migrated, or belonging to an ethnic minority was associated with less favourable outcome values in each of the two studies. In both cases, the effect of the social position on diabetes prevalence and blood glucose levels was mainly or even completely attributable to additive main effects of those dimensions, while intersectional effects played a subordinate or no role. In agreement with these findings, our results suggest that multiplicative intersectional effects play a smaller role in the risk of developing diabetes compared to additive effects of individual social determinants. In this way, the present study illustrates how taking an intersectional perspective, through the application of an intersectionality-informed approach such as MAIHDA, allows for a nuanced understanding of how social dimensions shape health outcomes. Hence, investigating the interplay of social dimensions through additive and intersectional effects enables a holistic approach to addressing health inequalities and developing tailored public health interventions.

In terms of practical implications, the results of the present study emphasize the need to consider the relevant impact of social position on the risk of developing type 2 diabetes also when planning and implementing prevention measures. In view of limited resources for prevention and health promotion, this raises the question of how precisely interventions to prevent diabetes should be tailored to particular groups. To address social disparities in health, Marmot et al. [54] recommend universal strategies, adjusting attention and intensity based on need. According to our findings, it is mainly additive effects of social determinants that explain social disparities in diabetes risk, whereas intersectional effects had a comparatively minor impact. We applied the MAIHDA approach to investigate whether certain intersectional strata show an increased risk for type 2 diabetes, suggesting a special need for prevention. However, in the context of type 2 diabetes risk, complex interactions of social dimensions seemed less significant. Our study did not reveal any intersectional strata with risk scores beyond what would be expected from the additive main effects. Consequently, preventive interventions for type 2 diabetes should prioritize target groups following significant main effects, while particularly addressing population groups at the intersection of disadvantaged social characteristics. This suggests considering the needs of males and those with low levels of education, with special attention to those for whom both apply.

The need for tailored prevention strategies is underscored by available evidence suggesting that both men and persons with low educational level might not benefit sufficiently from existing diabetes prevention measures. For instance, they are less likely to take up preventive services such as health check-ups [55, 56] or programs aimed at promoting healthy lifestyles and behaviour change [57–59]. In view of these findings, previous studies have successfully pioneered gender-sensitive interventions, such as lifestyle programs aimed at increasing physical activity in overweight men through their followership of their favourite soccer or hockey clubs, yielding positive effects in terms of weight loss [60]. In this sense, further investigation into mediating variables and processes linked to differences in diabetes risk and prevention in relation to sex/gender as well as level of education is needed. This could provide valuable insights to inform the development of tailored interventions accordingly.

### Strengths and limitations

To our knowledge, this is the first study examining preventive potential concerning type 2 diabetes within an intersectional framework in persons without known diabetes in Germany. We applied the MAIHDA approach, as it is considered a valuable tool to quantitatively investigate intersectional health inequalities, providing several advantages compared to single-level models [30]. This novel approach aims to descriptively explore health inequality across combinations of the considered social dimensions by mapping predicted outcomes simultaneously for all strata representing these combinations. A notable strength of MAIHDA lies in its alignment with the perspective of intersectional theory. Rather than reproducing notions of privilege and marginalization by examining possible intersectional effects as deviations from a reference group, MAIHDA enables the simultaneous analysis of possible intersectional effects in all intersectional strata. This is done by examining them as deviations of predicted strata values from expected values based on additive main effects [26]. In this way, also the effects of social positions combining social dimension characteristics of privilege and disadvantage can easily be mapped. Finally, MAIHDA models permit more social dimensions or corresponding categories and multiple resulting combinations to be included in the analysis while still being parsimonious and comparatively easy to interpret, whereas single-level models require the inclusion of a geometrically growing number of interaction terms [26].

Despite these strengths, we must address some limitations. The study sample was obtained using well-established sampling procedures for health telephone surveys to collect a population-based sample that allows to draw generalizable conclusions on health behaviour and risk factors for diabetes to the German-speaking population [34]. As the response rate was rather low, we included a survey-specific weighting factor in all analyses to compensate for deviations in the study sample compared to the resident population in Germany. Nevertheless, it is possible that the representativeness of the sample is limited regarding certain characteristics, since, for example, the proportion of people with a history of migration is lower than in the general German population which may be due to recruitment criteria like German language skills. As a considerable number of persons with a history of migration might not have had sufficient proficiency in German language to participate in the survey, the representativeness of the included individuals with migration history is likely to be reduced in comparison to this group in general population. What may further limit the interpretation of results on migration history is the average age difference between participants with and without history of migration. While in line with the situation in the German population, the sensitivity analysis controlling for age no longer showed a fixed effect of migration history on diabetes risk which suggests a confounding with age. However, calculating the diabetes risk score without considering age as a score component was not feasible, as there is no corresponding validation. This challenge stems from the fact that type 2 diabetes risk was calculated by means of a validated score based on diabetes-related risk factors, including age, to estimate the risk in individuals not yet affected and therefore still eligible for prevention, rather than relying on assessments by a physician or biomarkers. Although the GDRS has shown excellent discriminatory accuracy for predicting a type 2 diabetes diagnosis within 5 years, the assignment of points might be flawed since score components were assessed by self-report. Besides, the GDRS has primarily been validated for the resident population in Germany as a whole. As risk patterns in the development of diabetes may differ in persons with a history of migration, the GDRS may not reflect the diabetes risk of these individuals in the same way. It would thus be valuable to investigate the validity of the GDRS for this population group in future research.

Moreover, the exclusion of participants with diabetes according to the self-report of a diabetes diagnosis cannot rule out the possibility that the sample included people with an unreported or not yet diagnosed diabetes. Finally, the number of social dimensions and associated characteristics that could be investigated was limited in this study to ensure an adequate number of observations within each intersectional stratum. To further take advantage of the strengths of the MAIHDA approach, future studies that can rely on larger case numbers should also consider other potentially diabetes-related social determinants, such as area deprivation [61] or living arrangements [62]. In addition, further differentiation should be made between relevant subgroups, for example by regions of origin for persons with a history of migration.

## Conclusions

Mapping social disparities in the risk of developing type 2 diabetes, we found substantial differences in diabetes risk in Germany based on social position. These differences in diabetes risk seem to be primarily explained by additive main effects of the social dimensions that define social position, particularly sex/gender and education level, rather than intersectional effects. Therefore, to address the identified disparities in diabetes risk, it is recommended to implement targeted prevention measures adjusted to the needs of groups at increased risk, such as men and individuals with low educational level. To this end, further research on social inequality in relation to type 2 diabetes should explore underlying processes and intersectional patterns to inform effective prevention measures and reduce the risk of type 2 diabetes in vulnerable populations.

### Electronic supplementary material

Below is the link to the electronic supplementary material.

Supplementary Material 1

Supplementary Material 2

## Data Availability

The data that support the findings of this study are available from the Research Data Centre at the Robert Koch Institute (RKI) (e-mail: fdz@rki.de), but restrictions apply to the availability of these data, which were used under license for the current study, and so are not publicly available. Data are however available upon reasonable request from the authors, who will forward the request to the RKI Research Data Centre, which must grant permission.

## Abbreviations

BZgA: German Federal Centre for Health Education
CASMIN: Comparative Analysis of Social Mobility in Industrial Nations
DIfE: German Institute of Human Nutrition Potsdam-Rehbrücke
GDRS: German Diabetes Risk Score
ICC: Intraclass correlation
MAIHDA: Multilevel analysis of individual heterogeneity and discriminatory accuracy
PCV: Proportional change in variance
REML: Restricted maximum likelihood
RKI: Robert Koch Institute
VPC: Variance partition coefficient
