# Supplementary material for: Intersectional analysis of social disparities in type 2 diabetes risk among adults in Germany: results from a nationwide population-based survey
Source: BMC Public Health. 2024 Feb 16;24:498. doi: 10.1186/s12889-024-17903-5 (PMC10874065; doi:10.1186/s12889-024-17903-5)
Supplement: Supplementary file 1 — Supplementary Material 1 [file 12889_2024_17903_MOESM1_ESM.docx]

## Additional file 1: Table S1.

Results from the sensitivity analysis are given in Table S1, i.e., parameter estimates from MAIHDA intersectional models for diabetes risk score including age in years as a continuous control variable.

Table S1. Parameter estimates from MAIHDA intersectional models for diabetes risk score controlled for age (n=2253)

|  | **Model 1. Simple intersectional model** | | **Model 2. Intersectional interaction model** | |
| --- | --- | --- | --- | --- |
|  | **Estimate** | **95% CI** | **Estimate** | **95% CI** |
| **Fixed effects** |  |  |  |  |
| Intercept | 1.79 | (-1.13, 4.71) | -4.27 | (-6.99, -1.61) |
| Age | 0.76 | (0.73, 0.79) | 0.76 | (0.73, 0.80) |
| Sex/Gender |  |  |  |  |
| Female (reference) |  |  | - | - |
| Male |  |  | 6.80 | (5.08, 8.54) |
| History of migration |  |  |  |  |
| No history of migration (reference) |  |  | - | - |
| History of migration |  |  | 1.28 | (-0.68, 3.18) |
| Educational level (CASMIN) |  |  |  |  |
| High (reference) |  |  | - | - |
| Middle |  |  | 2.60 | (0.60, 4.64) |
| Low |  |  | 3.71 | (1.49, 6.00) |
| **Measures of variance** |  |  |  |  |
| Between-strata variance | 14.70 | (4.58, 32.04) | 0.83 | (0.00, 5.07) |
| Within-strata variance | 100.6 | (92.47, 108.35) | 100.66 | (92.52, 108.42) |
| VPC (%) | 12.75 |  | 0.82 |  |
| PCV (%) |  |  | 93.55 |  |
| 1-PCV (%) |  |  | 6.45 |  |

95% CI = 95% confidence intervals; PCV = proportional change in the between-strata variance; VPC = variance partition coefficient.
