# Supplementary material for: Intersectional analysis of social disparities in type 2 diabetes risk among adults in Germany: results from a nationwide population-based survey
Source: BMC Public Health. 2024 Feb 16;24:498. doi: 10.1186/s12889-024-17903-5 (PMC10874065; doi:10.1186/s12889-024-17903-5)
Supplement: Supplementary file 2 — Supplementary Material 2 [file 12889_2024_17903_MOESM2_ESM.docx]

Additional file 2: Figure S1.

An adjusted version of Figure 2 is given in Figure S1 including a labelling grid. Stratum-level residuals regarding GDRS obtained from model 2 are depicted with 95% confidence intervals sorted by stratum ID.


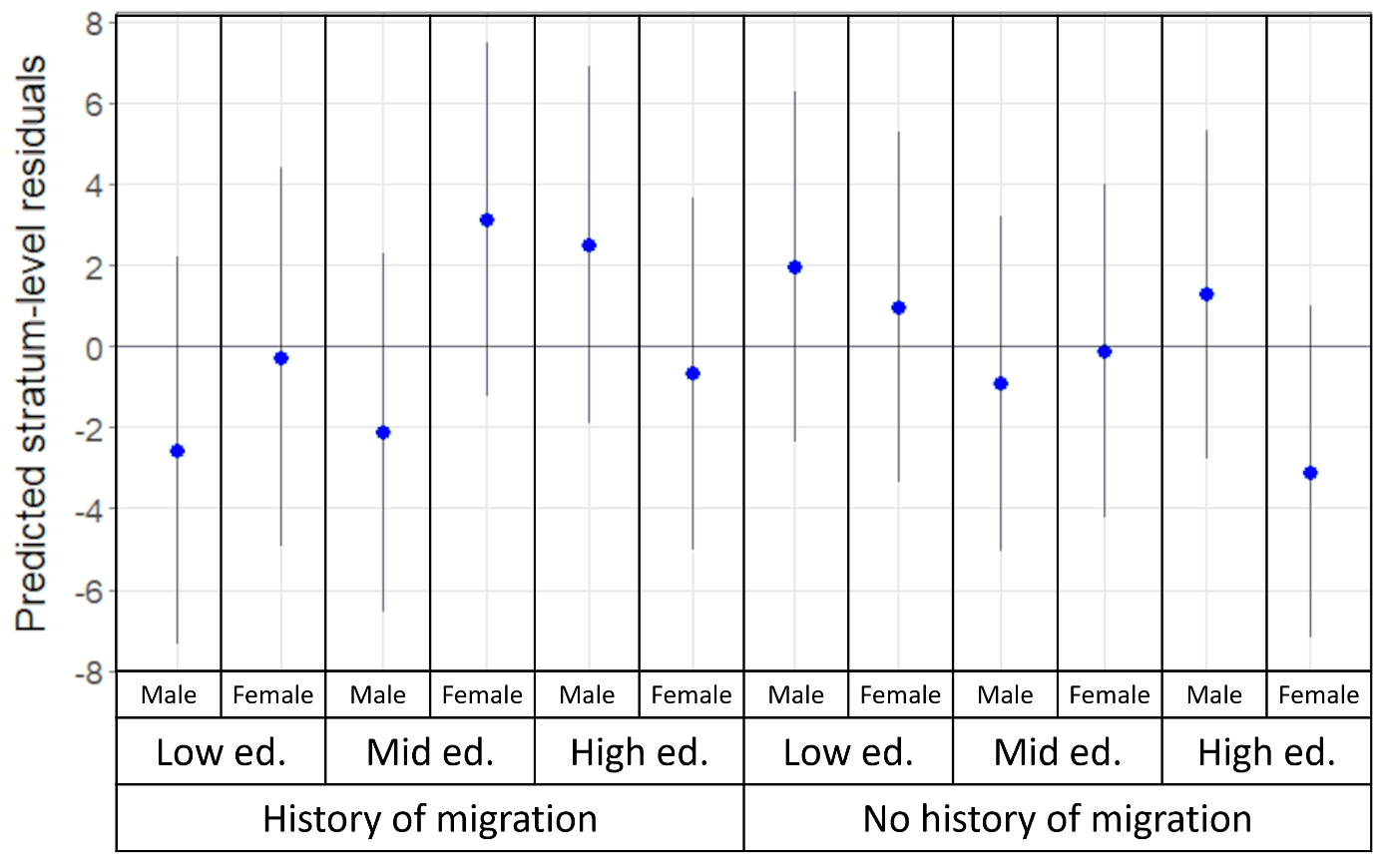


Figure S1. Predicted stratum-level residuals regarding GDRS with 95% confidence intervals obtained from model 2 sorted by stratum ID. ed.: educational level (See Figure 2 for a version sorted in ascending order of rank)
